# CMDMamba: dual-layer Mamba architecture with dual convolutional feed-forward networks for efficient financial time series forecasting

**DOI:** 10.3389/frai.2025.1599799

**Published:** 2025-07-15

**Authors:** Zhenkai Qin, Baozhong Wei, Yujia Zhai, Ziqian Lin, Xiaochuan Yu, Jingxuan Jiang

**Affiliations:** ^1^Network Security Research Center, Guangxi Police College, Nanning, China; ^2^School of Information Technology, Guangxi Police College, Nanning, China; ^3^School of Computer Science and Artificial Intelligence, Southwest Jiaotong University, Chengdu, China; ^4^Institute of Software, Chinese Academy of Sciences, Beijing, China; ^5^School of Public Administration, Guangxi Police College, Nanning, China; ^6^School of Business Administration, Guangxi Vocational and Technical Institute of Industry, Nanning, Guangxi, China

**Keywords:** Mamba, financial time series forecasting, deep learning, State Space Models, computational efficiency

## Abstract

**Introduction:**

Transformer models have demonstrated remarkable performance in financial time series forecasting. However, they suffer from inefficiencies in computational efficiency, high operational costs, and limitations in capturing temporal dependencies.

**Methods:**

To address these challenges, we propose the CMDMamba model, which is based on the Mamba architecture of state-space models (SSMs) and achieves near-linear time complexity. This significantly enhances the real-time data processing capability and reduces the deployment costs for risk management systems. The CMDMamba model employs a dual-layer Mamba structure that effectively captures price fluctuations at both the micro- and macrolevels in financial markets and integrates an innovative Dual Convolutional Feedforward Network (DconvFFN) module. This module is able to effectively capture the correlations between multiple variables in financial markets. By doing so, it provides more accurate time series modeling, optimizes algorithmic trading strategies, and facilitates investment portfolio risk warnings.

**Results:**

Experiments conducted on four real-world financial datasets demonstrate that CMDMamba achieves a 10.4% improvement in prediction accuracy for multivariate forecasting tasks compared to state-of-the-art models.

**Discussion:**

Moreover, CMDMamba excels in both predictive accuracy and computational efficiency, setting a new benchmark in the field of financial time series forecasting.

## 1 Introduction

Financial time series forecasting constitutes a fundamental component of quantitative finance, underlying essential tasks such as algorithmic trading, risk assessment, and portfolio optimization (Liu and Kim, 2025). Accurate and robust predictive models enable market participants to anticipate asset price dynamics, adapt to structural market changes, and design data-driven investment strategies (Salinas et al., 2020). However, financial time series exhibit distinctive complexities absent in conventional domains (e.g., energy demand, traffic flow), including pronounced nonstationarity, high frequencystochastic noise, regime-switching behavior, and evolving cross-asset dependencies driven by macroeconomic indicators, geopolitical shocks, and behavioral factors (Patel and Singh, 2023). These features significantly complicate the modeling process and undermine the effectiveness of traditional time series techniques.Conventional statistical models such as Autoregressive Integrated Moving Average (ARIMA) (Pokou et al., 2024) and Generalized Autoregressive Conditional Heteroskedasticity (GARCH) (Han et al., 2024) have been widely applied in financial forecasting. However, their reliance on linear assumptions and limited capacity to adapt to dynamic structural changes render them inadequate for disentangling meaningful market signals from pervasive noise, especially under high-volatility conditions (Sezer et al., 2020).

To address these limitations, alternative architectures such as Multi-Layer Perceptrons (MLPs) and Temporal Convolutional Networks (TCNs) have been explored. MLP-based models offer advantages in terms of linear computational complexity and robustness to low signal-to-noise ratios, largely attributed to their residual design mechanisms (Zeng et al., 2022). However, they generally lack explicit temporal modeling capabilities and tend to respond inadequately to abrupt time-series fluctuations. In contrast, TCNs utilize dilated causal convolutions to effectively capture long-range dependencies while maintaining temporal causality (Ebrahimpour et al., 2011), making them well-suited for data with periodic or trend-based patterns. Nevertheless, TCNs often struggle with modeling nonlinear dynamics and adapting to external perturbations (Behera et al., 2023; Lara-Benítez et al., 2020).

Recent advances in State Space Models (SSMs) (Triantafyllopoulos, 2021; Behrouz et al., 2024a), particularly the Mamba architecture (Gu and Dao, 2023), have introduced a new paradigm in sequence modeling by combining selective state transitions with linear time complexity to effectively capture long-range dependencies and suppress noise. However, when applied to financial time series forecasting, Mamba reveals key limitations: it struggles to model hierarchical intra-sequence structures, making it difficult to distinguish short-term fluctuations from long-term economic patterns, and it lacks mechanisms for capturing complex inter-variable dependencies vital to multivariate financial systems. These shortcomings highlight the need for enhanced architectures that build on Mamba's strengths while incorporating hierarchical feature extraction and variable-wise interaction modeling to improve forecasting performance in volatile and interconnected financial environments.

To bridge these gaps, we propose CMDMamba, a novel architecture tailored for financial time series forecasting. CMDMamba integrates a dual-layer Mamba framework with a hierarchical channel-aware learning mechanism to capture the multi-scale temporal structures intrinsic to financial data. The first Mamba layer is tuned for high responsiveness to short-term fluctuations, enabling precise identification of micro-level movements, while the second layer targets long-term dependencies that reflect underlying structural trends. To further enhance inter-variable interaction modeling, we introduce a Dual Convolutional Feedforward Network (DConvFFN), which preserves temporal locality and strengthens cross-channel information flow. Additionally, a block-wise sequence partitioning strategy is adopted to increase semantic granularity, thereby improving the model's capability in capturing nuanced temporal transitions and enhancing generalization performance. The principal contributions of this study are as follows:

- We propose a novel architectural framework that extends state space modeling to the domain of financial time series forecasting. By leveraging the structural advantages of the Mamba architecture, this framework effectively addresses the challenges posed by high volatility and complex feature dependencies inherent in financial data.
- Extensive experiments conducted on four real-world financial datasets demonstrate that CMDMamba achieves an average improvement of 10.4% in multivariate forecasting accuracy compared to state-of-the-art benchmarks. The model excels under high-noise and high-volatility conditions, highlighting its robustness and practical relevance.
- The proposed framework achieves superior balance between efficiency and performance, maintaining the computational efficiency advantages of linear complexity while matching or surpassing state-of-the-art Transformer-based models in predictive accuracy.

## 2 Related work

### 2.1 Financial time series forecasting

Significant advances have been witnessed in the financial time series forecasting domain, which are driven by deep learning architectures (Ge et al., 2022; Wu et al., 2024; Torres et al., 2021). Despite the notable advancements achieved by deep learning models such as Transformers and Temporal Convolutional Networks (TCNs) in financial time series forecasting, significant challenges remain in fully capturing the inherent complexity of financial data (Khan and Khan, 2024; Smith and Doe, 2024). For instance, Transformer models suffer from quadratic computational complexity, rendering them inefficient for processing complex financial data. Although TCNs are effective in modeling long-range dependencies, they struggle with capturing nonlinear dynamics and adapting to exogenous shocks. Recently, State Space Models (SSMs) have attracted increasing attention for their strengths in long-sequence modeling (Nguyen and Chen, 2025). However, existing SSMs–such as Mamba–still fall short in capturing the hierarchical structure and cross-variable interactions that are critical in financial time series (Kumar and Lee, 2023; Zhang and Wang, 2024). These limitations form the theoretical foundation for the development of CMDMamba. However, three fundamental challenges still remain: nonstationary data distributions, ultrahigh-frequency noise contamination, and nonlinear cross-asset dependencies. Traditional linear statistical models and their regularized variants, although maintaining computational efficiency and interpretability through shrinkage techniques, are essentially restricted by their dependence on linear additive assumptions Johnson and Martinez (2023). This limitation seriously limits their ability to capture the intricate non-linear dynamics that is inherent in financial markets (Luo et al., 2021). Empirical studies have shown that these methods have crucial deficiencies in modeling sudden regime shifts in complex financial situations.

Innovations in deep learning architectures (Zhang and Hua, 2025; Yu et al., 2024) have brought about a profound transformation in the methodological framework for financial prediction. Architecturally, multilayer perceptron (MLP)—based systems have improved gradient propagation. They achieve this through stacked fully - connected layers with residual connections, as demonstrated by DeepAR's excellent performance in low—frequency return prediction benchmarks (Salinas et al., 2020). Temporal convolutional networks (TCNs) utilize causal and dilated convolution operations to build exponentially expanding temporal receptive fields (Yao et al., 2023). Researchers have achieved significant improvements in high—frequency trade signal detection by stacking multi—layer convolutional modules, especially in market microstructure analysis (Hao and Gao, 2020). To overcome the quadratic complexity bottleneck in Transformer—based sequence processing, optimized solutions incorporating sparse attention mechanisms have been developed. The Informer (Zhou et al., 2021) architecture uses probabilistic sampling strategies. Although this approach greatly reduces computational resource demands, the remaining complexity is still not practical for real—world financial systems. Despite the use of enhanced techniques leveraging time—frequency domain transformations (such as fast Fourier transforms) (Qin et al., 2025), spectral analysis capabilities are still insufficient when dealing with ultra—low—frequency signals contaminated by strong noise (Zhou et al., 2022).

Current methodologies continue to exhibit critical deficiencies in modeling dynamic coupling relationships among multivariate series. The dual constraints of computational complexity and prediction accuracy present urgent challenges for real-time deployment, particularly pronounced in high-frequency trading environments, where the synergistic mechanisms between market microstructure noise and macroeconomic policy shocks remain insufficiently resolved (Song et al., 2024; Chomicz-Grabowska and Orlowski, 2020). CMDMamba adopts the Mamba architecture, which ingeniously reduces computational complexity to a linear level. The model also introduces a dual-layer Mamba design, dynamically adjusting the state decay rate according to different sensitivities to microstructure and macroeconomics, thereby effectively weakening noise interference and significantly enhancing overall model performance.

### 2.2 Applications of State Space Models

State Space Models (SSMs) (Gu et al., 2021a,d) have experienced a renaissance in sequential modeling, primarily attributed to the groundbreaking advancements in Structured State Space Sequence Models (S4) (Gu et al., 2021e,b). The innovative High-Order Polynomial Projection Operators (HiPPO) Gu et al. (2020) initialization framework established by S4 has created a new paradigm, demonstrating unprecedented capabilities in long-range dependency modeling. Subsequent studies have continuously enhanced computational efficiency while preserving the theoretical foundation of continuous system modeling. Notably, the diagonal parameterized SSMs (S4D) Gu et al. (2022) and data-dependent SSMs (DSS) have laid crucial groundwork for modern SSM applications across diverse domains (Gu et al., 2021c).

The emergence of the Mamba architecture has catalyzed a paradigm shift in this field, representing a quantum leap in SSM research. This seminal work innovatively incorporates time-varying parameterized state matrices combined with hardware-aware parallel scanning algorithms. This dual innovation achieves linear computational complexity while maintaining global dependency capture capabilities, attaining positional awareness levels comparable to Transformer architectures. Such breakthroughs have precipitated the proliferation of derivative models, finding extensive applications in language modeling, audio processing, computer vision, and temporal sequence analysis. Notwithstanding these advancements, current SSM architectures confront substantial optimization challenges. The S-Mamba (Wang et al., 2025) model, while implementing decoupled modeling through channel-temporal state space tensor decomposition (Feng et al., 2022), theoretically exhibits deficiencies in dynamically representing high-order cross-feature interactions within temporal data. The bidirectional gating mechanism in MambaMixer (Behrouz et al., 2024b), though capable of synchronously capturing inter-sequence and intra-sequence dependencies, suffers from significant information loss during forward-backward feature selection processes. TimeMachine's (Ahamed and Cheng, 2024) quad-scale architecture integrates channel mixing mechanisms, yet its static statistical feature-based policy selector proves inadequate in adapting to distributional drift phenomena inherent in non-stationary time series. The parameter-sharing scheme in Bi-Mamba (Liang et al., 2024) enhances temporal modeling efficiency but introduces bidirectional state coupling interference. Moreover, its reverse scanning mechanism requiring full-sequence input conflicts with the low-latency demands of streaming inference, creating critical deployment barriers that highlight systemic architectural contradictions in balancing temporal correlation and real-time processing.

Despite notable progress, critical scientific challenges remain unresolved: how to develop novel architectures capable of capturing cross-variable correlations, modeling complex nonlinear interactions, and maintaining linear computational complexity. This bottleneck constrains the applicability of the Mamba-series models in complex scenarios such as financial forecasting and industrial IoT. Our proposed DconvFFN module effectively captures these correlations, thereby enhancing the predictive performance of the model.

## 3 Methodology

### 3.1 Problem definition

Financial time series forecasting is formally defined as follows. Let $X^{*}=\left\{x_{1},x_{2},\ldots ,x_{N}\right\}$ denote a multivariate financial time series, where each observation $x_{t}\in {\mathbb{R}}^{d}$ represents a *d*-dimensional vector of financial variables at time step *t*. Given a fixed-length lookback window *T*, the forecasting task at any time point *t* involves predicting the future τ-step sequence $X_{t+1:t+\tau }=\left\{x_{t+1},x_{t+2},\ldots ,x_{t+\tau }\right\}$ based on the historical observations within the window $X_{t-T+1:t}=\left\{x_{t-T+1},x_{t-T+2},\ldots ,x_{t}\right\}$. This formulation captures the temporal dependencies and multivariate interactions inherent in financial data, enabling the prediction of future trends and patterns.

### 3.2 State Space Models

State Space Models (SSMs) describe recurrent processes through latent states **h**(*t*) ∈ ℝ^*N*^ governed by a first-order differential equation. Inputs **x**(*t*) ∈ ℝ^*D*^ drive state evolution, while outputs **y**(*t*) ∈ ℝ^*M*^ are generated via a mapping from **h**(*t*). The dynamic process is illustrated in Equation 1:


(1) $$\begin{array}{l} {\text{h}}^{\prime }\left(t\right)=\text{A}\text{h}\left(t\right)+\text{B}\text{x}\left(t\right),\\ \text{y}\left(t\right)=\text{C}\text{h}\left(t\right) \end{array}$$

where **A** ∈ ℝ^*N*×*N*^ denotes the state transition matrix governing temporal dynamics, **B** ∈ ℝ^*N*×*D*^ the input projection matrix, and **C** ∈ ℝ^*M*×*N*^ the output projection matrix. All three matrices are learnable parameters optimized during training. By employing zero-order hold (ZOH) techniques, the SSM can be discretized, as shown in Equations 2:


(2) $$\begin{array}{l} {\text{h}}_{t}=\bar{\text{A}}{\text{h}}_{t-1}+\bar{\text{B}}{\text{x}}_{t},\\ {\text{y}}_{t}=\text{C}{\text{h}}_{t} \end{array}$$

where the state matrix $\bar{\text{A}}$ and input matrix $\bar{\text{B}}$ are derived from continuous parameters (**A**, **B**, Δ*t*) through a discretization method. The specific transformation formulas (Equation 3) are given as follows:


(3) $$\begin{array}{l} \bar{\text{A}}=\exp\left(\Delta t\text{A}\right),\\ \bar{\text{B}}={\left(\Delta t\text{A}\right)}^{-1}\left(\exp\left(\Delta t\text{A}\right)-\text{I}\right)\cdot \Delta t\text{B} \end{array}$$

Where the invertibility of Δ*t***A** enables efficient discretization through linear recurrence operations, the Structured State Space model (S4) introduces HiPPO-initialized constraints on matrix **A**. This integration establishes S4 as a computationally efficient hybrid architecture that combines classical state-space formulations with deep learning principles, achieving superior long-range sequence modeling capabilities compared to conventional recurrent networks.

**Figure 1:**
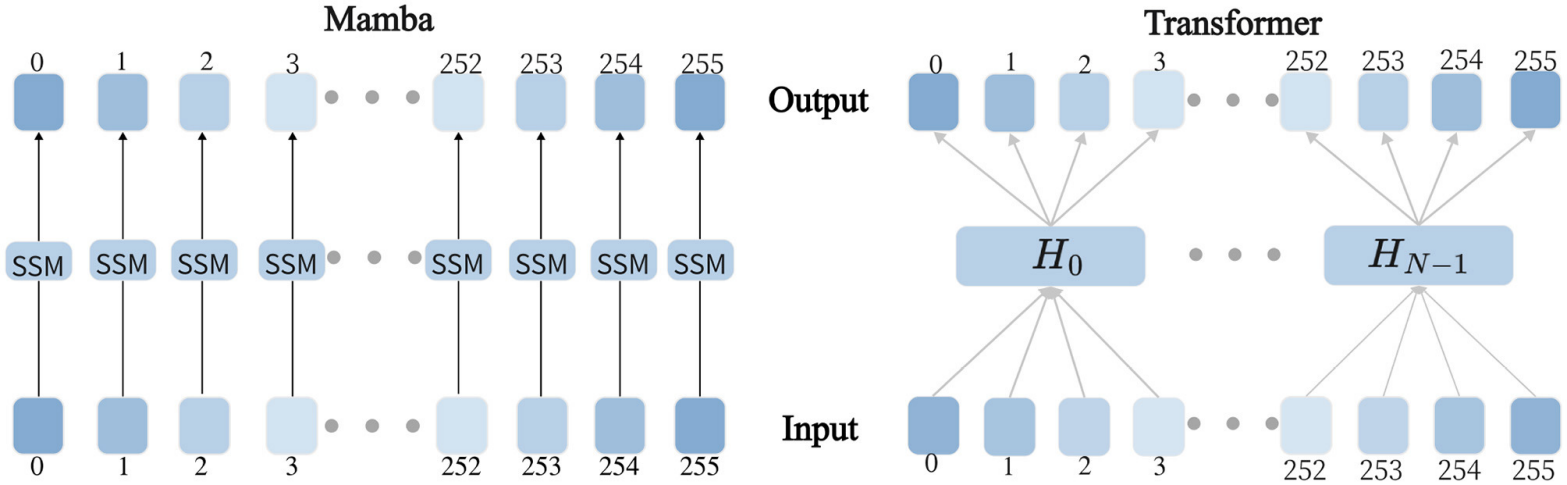
Mamba and Transformer Modeling Process Diagram, where the **left** figure illustrates the modeling process of Mamba, and the **right** figure illustrates the modeling process of Transformer.

Mamba innovatively employs a data-driven approach to parameterize critical matrices while integrating a dynamic selection mechanism into the S4 architecture. As illustrated in Figure 1, the proposed hardware-aware parallel algorithm significantly enhances training efficiency. In contrast to self-attention-based Transformer models, this architecture achieves linear computational complexity while preserving global receptive fields. This breakthrough enables the model to efficiently capture long-range dependencies in time series forecasting without incurring the high computational costs associated with conventional methods, thus achieving an optimal balance between computational efficiency and temporal modeling capability.

### 3.3 Model architecture

**Figure 2:**
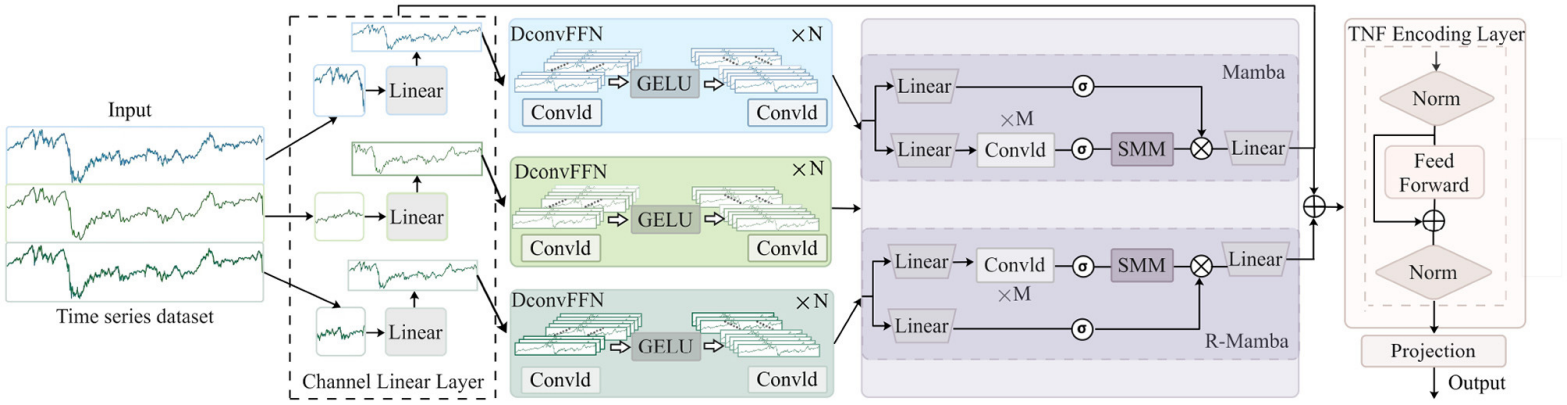
CMDMamba consists of four modules: the Channel Linear Layer module, which uses a linear embedding mechanism for variable decoupling, the DconvFFN module for decoupled learning of multi-dimensional features, the Double-Layer Mamba module for capturing short-term fluctuations and long-term dependencies, and the TNF Encoding Layer for enhancing the convergence and training stability of deep networks.

The architecture of the CMDMamba model, as illustrated in Figure 2, comprises four critical processing stages: Initially, the embedding layer divides the input time-series data into feature blocks with high information density. Subsequently, the DconvFFN module models local correlations among features to achieve dynamic fusion of multi-feature representations. Then, a dual cascaded Mamba processing module is designed, where the high-sensitivity Mamba block captures microscopic fluctuation characteristics within sequences, while the low-sensitivity Mamba block focuses on modeling long-term dependencies across time steps. Finally, the TNF Encoding module blends information from the Mamba-processed temporal features, generating a final feature vector with robust representational capabilities.

#### 3.3.1 Channel linear layer

The embedding layer of the proposed model is designed to handle multivariate time series input data *X*_*in*_, with its core architecture inspired by the iTransformer (Liu et al., 2023a) framework through the adoption of a variable-disentangled linear embedding mechanism. This design achieves refined modeling of temporal patterns by independently processing chronological features from individual variables. Furthermore, a variable-specific linear embedding layer is established to separately consider each variable's characteristics during encoding. This strategic separation effectively mitigates noise induced by variable mixing while temporally preserving intrinsic sequential relationships, thereby enhancing semantic information representation. Subsequently, the encoded features of each variable are projected into a high-dimensional space through linear transformation, where *D* denotes the embedding dimension, as shown in Equation 4:


(4) $$\begin{array}{l} X=\text{Linear}\left(\text{Batch}\left(X_{in}\right)\right) \end{array}$$

#### 3.3.2 DconvFFN

To enhance the multidimensional feature extraction capability in financial time series forecasting, we innovatively propose the DconvFFN module. Addressing three critical limitations of traditional single-convolutional layers—restricted feature representation capacity, inadequate cross-variable dependency modeling, and the trade-off between computational efficiency and performance—our module achieves multidimensional feature decoupling learning through synergistic integration of two functionally complementary 1D convolutional layers. Formally, given an input tensor *X* ∈ ℝ^*B*×*V*×*D*^ (where *B* denotes batch size, *V* the number of variables, and *D* the feature dimension), the DconvFFN operation is formulated as Equation 5:


(5) $$\begin{array}{l} \;\text{DconvFFN}\left(X\right)=\text{Dropout}\\ \left(W_{2}*\text{GELU}\left(\text{Dropout}\left(W_{1}*X+b_{1}\right)\right)+b_{2}\right) \end{array}$$

The first convolutional layer $W_{1}\in {\mathbb{R}}^{K_{1}\times V\times d}$ operates along the variable dimension with learnable local receptive fields (kernel size *K*_1_), performing feature re-encoding for each variable's temporal sequence to generate intermediate representations with expanded dimension *d*. Subsequently, the second convolutional layer $W_{2}\in {\mathbb{R}}^{K_{2}\times d\times D}$ conducts cross-variable aggregation (kernel size *K*_2_) in the expanded feature space, employing a dynamic weight-sharing mechanism to capture nonlinear interaction patterns among variables. The coordinated use of dual Dropout layers and GELU activation function ensures model generalization while enabling nonlinear feature information flow with biased filtering.

#### 3.3.3 CMDMamba layer

CMDMamba's basic building block is the Double-Layer Mamba module. It employs two parallel Mamba modules with different temporal sensitivities to jointly process sequential data, thereby capturing both short-term fluctuations and long-term dependencies. Consider an input tensor ***x*** ∈ ℝ^*B*×*V*×*D*^ fed into a single Mamba module, where *B* represents the batch size, *V* is the number of variables, and *D* denotes the hidden dimension. The computational process can be divided into three key stages, with the first stage shown in Equation 6:


(6) $$\begin{array}{l} x^{\prime }=\text{SiLU}\left(\text{Conv1D}\left(\text{Linear}\left(x\right)\right)\right),\\ z=\text{SiLU}\left(\text{Linear}\left(x\right)\right) \end{array}$$

The Sigmoid Linear Unit (SiLU) activation, as described in [3] and also known as Swish, introduces non-linearity. The linear transformation Linear(·) projects the input into distinct feature spaces, while the depthwise 1D convolution Conv1D(·) conducts local temporal filtering to enhance the discovery of short-term patterns. This design allows ***x***′to focus on localized contextual features, while **z** retains global signal characteristics, as shown in Equation 7:


(7) $$\begin{array}{l} y^{\prime }=\text{Linear}\left(\text{SelectiveSSM}\left(x^{\prime }\right)⊗z\right),\\ y=\text{LayerNorm}\left(y^{\prime }+x\right) \end{array}$$

The SelectiveSSM(·) implements an input-adaptive state-space model with dynamic parameterization mechanisms. These mechanisms adjust the memory decay rates based on the local context ***x***′. The element-wise multiplication ⊗ creates a gating mechanism that selectively combines the temporal dynamics from both branches. The Dual-layer Mamba deploys two parallel Mamba modules with different temporal sensitivities: the high-sensitivity branch focuses on capturing rapid local variations, generating output *Y*_1_, while the low-sensitivity branch models the slowly evolving global patterns, producing output *Y*_2_. The final output is obtained through feature aggregation, as shown in Equation 8:


(8) $$\begin{array}{l} Y=Y_{1}+Y_{2}. \end{array}$$

Outputs of the two Mamba blocks, *Y*_1_ and *Y*_2_, are aggregated to yield the final output *Y* of the double-layer Mamba block.

## 4 Experiment

### 4.1 Datasets

**Figure 3:**
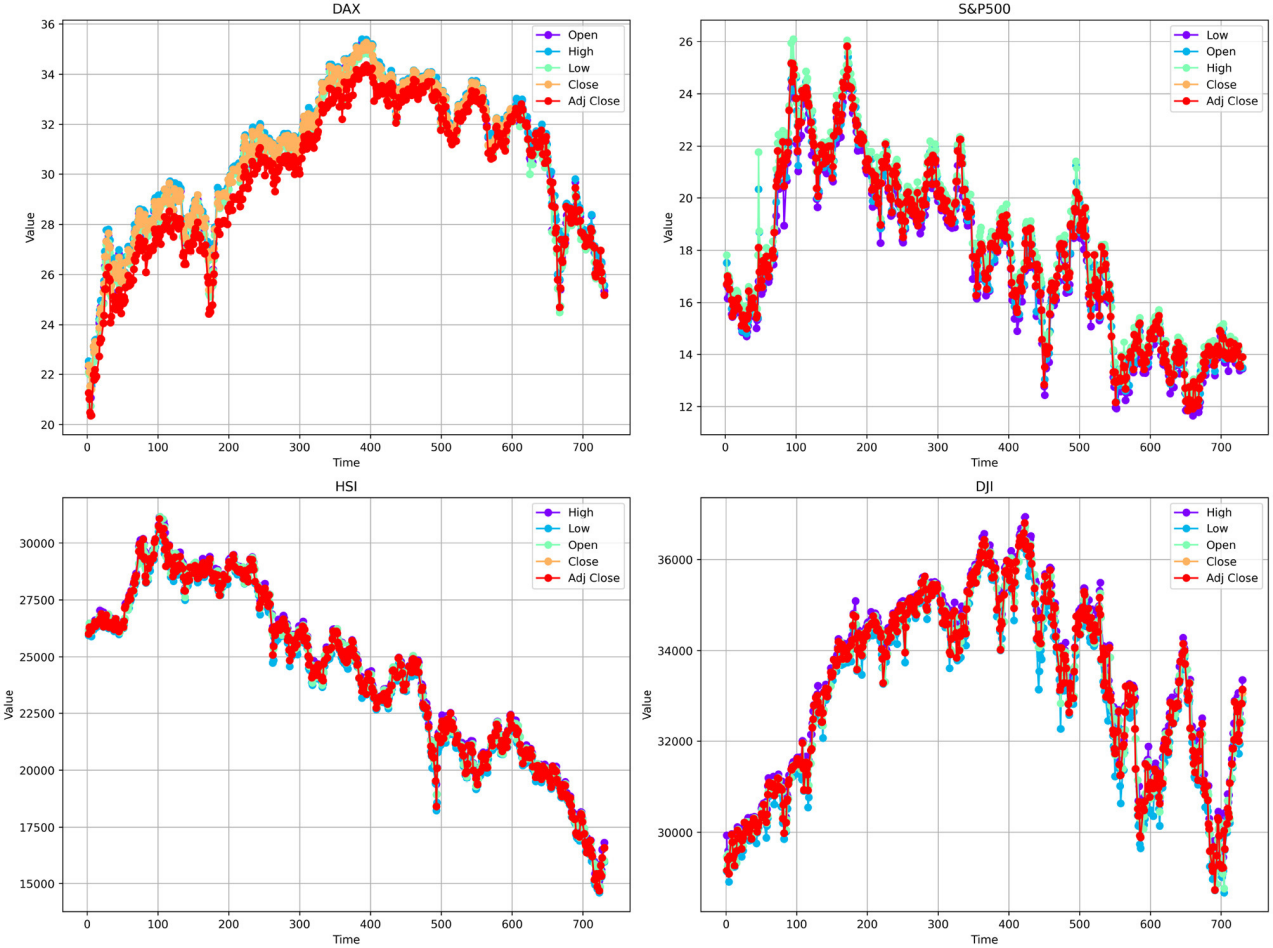
Descriptive statistics of various features across four different datasets.

In this experiment, to evaluate the model performance, we selected four publicly available financial time series datasets. These datasets encompass historical trading data from diverse financial markets and were chosen based on their broad applicability and the significant challenges they present in the financial domain. Specifically, these data sets include the German DAX index, the Dow Jones Industrial Average (DJI), the Hang Seng Index (HSI), and Standard & Poor's 500 Index (S&P500). All data sets cover the period from January 2, 2014, to December 12, 2022, with daily observations collected throughout this timeframe. Each dataset was partitioned into training, validation, and test sets using a 7:1:2 ratio. Detailed characteristics of the data sets are provided in Table 1, In addition, Figure 3 illustrates the price trends of various variables in each financial dataset over the past two years.

**Table 1:** Detailed characteristics of all datasets.

**Indicator**	**Indicator definition**	**Corresponding characteristics**
Date	Date eRecording day	Temporal characteristics
High	Highest trading price	
Low	Lowest trading price	
Open	Opening trading price	Price type
Close	Closing trading price	
Adj close	Adjusted closing price	
Volume	Total trading volume	Active trading

### 4.2 Implementation details

All experiments were conducted using the following configuration: an L2 loss function with Adam optimizer (Kingma and Ba, 2014), initial learning rate of 5 × 10^−5^, and batch size of 8. The Transformer-based architecture employed an attention factor of 3, while the Mamba-based model utilized a state expansion factor of 2. Weight decay was consistently set to 0.1 throughout all trials. Training procedures implemented early stopping with a patience period of 10 epochs. To ensure statistical reliability, each experimental condition was independently repeated three times with random initialization. The complete implementation, developed using PyTorch (Paszke et al., 2019), was executed on a dedicated NVIDIA Tesla V100 GPU (32GB memory) (Markidis et al., 2018) to maintain computational consistency across trials.

### 4.3 Evaluation indicators

In time series forecasting, the accuracy of the model is crucial for the reliability of decision-making. Mean squared error (MSE), mean absolute error (MAE), and mean absolute percentage error (MAPE) are commonly used evaluation metrics. They can quantify the deviation between the predicted values of the CMDMamba model and the actual observed values from different perspectives, providing intuitive data for evaluating the model's performance. As shown in Equation 9, the calculation is performed as follows:


(9) $$\begin{array}{l} \text{MSE}=\frac{1}{n}\overset{n}{\underset{i=1}{\sum }}{\left(y_{i}-{ŷ}_{i}\right)}^{2},\\ \text{MAE}=\frac{1}{n}\overset{n}{\underset{i=1}{\sum }}|y_{i}-{ŷ}_{i}|,\\ \text{MAPE}=\frac{1}{n}\overset{n}{\underset{i=1}{\sum }}|\frac{y_{i}-{ŷ}_{i}}{y_{i}}|\times 100\% \end{array}$$

In these formulas, *y*_*i*_ represents the actual observed value, ŷ_*i*_ is the predicted value of the model, and *n* denotes the total number of data points. To accurately compare model performance, we normalize the values. With metrics such as MSE and MAE, we can intuitively assess the prediction accuracy of the CMDMamba model. The lower the values of these two metrics, the closer the predicted values are to the actual values, indicating better prediction performance of the model.

### 4.4 Baselines

Since models applicable to general time series forecasting are equally suitable for financial time series prediction, we comprehensively adopt state-of-the-art models from the time series community as baseline references. These include transformer-based models: Autoformer (Wu et al., 2021), Informer Zhou et al. (2021), Reformer (Kitaev et al., 2020), Pyraformer (Liu et al., 2022b), Crossformer (Liu et al., 2023b), and iTransformer (Li et al., 2019); MLP-based models: TiDE (Das et al., 2023); linear-based models: DLinear (Zeng et al., 2023); convolution-based models: SCINet (Liu et al., 2022a); recurrent-based models: LSTM; Statistical modeling-based methods:GARCH; and Mamba-based models: S_Mamba (Shehzad et al., 2020).

### 4.5 Multivariate results

In multivariate predictive analysis, multiple time series are considered simultaneously to evaluate the model's ability to capture interdependencies and mutual influences among various features. The experimental results in four data sets demonstrate that CMDMamba consistently outperforms most baselines and prediction horizon configurations, as shown in Table 2. CMDMamba significantly reduced the mean squared error (MSE) by 11. 6% in DAX (0.570 → 0.504), 1. 5% in DJI (0.200 → 0.197), 0.4% in HSI ( 0.563 → 0.561), and 10.2% in S&P500 (0.498 → 0.447) compared to the previous best results. Compared to the previous best models, the performance of CMDMamba in this configuration improved by 10.4%. As forecast horizons expand, modeling complex long-range dependencies becomes increasingly demanding. In this context, the CMDMamba model distinguishes itself through its exceptional capability to efficiently integrate temporal information and intricate cross-variable dependencies. Its robust performance has been thoroughly validated across multiple real-world datasets, showcasing both outstanding prediction accuracy and broad applicability. However, the performance improvement of CMDMamba on the DJI and HSI datasets is relatively less pronounced compared to other datasets. This may be attributed to the fact that both datasets represent comprehensive stock indices characterized by relatively stable macro trends, rendering their temporal patterns less responsive to the dynamic state transition mechanisms that CMDMamba relies on.

**Table 2:** Multivariate forecasting results across four datasets under forecast horizons *O* ∈ {12, 36, 58, 96}.

**Model**		**Ours**			**S_Mamba**			**iTransformer**			**Film**			**Autoformer**			**Informer**			**LSTM**			**GARCH**		
**Metric**		**MSE**	**MAE**	**MAPE**	**MSE**	**MAE**	**MAPE**	**MSE**	**MAE**	**MAPE**	**MSE**	**MAE**	**MAPE**	**MSE**	**MAE**	**MAPE**	**MSE**	**MAE**	**MAPE**	**MSE**	**MAE**	**MAPE**	**MSE**	**MAE**	**MAPE**
DAX	**12**	**0.246**	**0.351**	1.182	0.283	0.374	1.405	0.285	0.376	1.665	0.624	0.607	2.870	0.633	0.616	1.985	1.170	0.904	1.158	0.890	0.739	1.069	1.033	0.775	**0.991**
	**24**	**0.413**	**0.464**	1.390	0.459	0.497	1.779	0.469	0.506	2.040	0.792	0.688	1.169	0.779	0.686	2.109	1.647	1.097	1.462	1.271	0.930	1.358	1.037	0.778	**0.994**
	**36**	**0.504**	**0.520**	1.321	0.570	0.566	1.697	0.576	0.569	1.964	1.016	0.787	1.581	0.912	0.751	1.492	2.055	1.224	1.420	2.356	1.324	1.204	1.039	0.780	**0.996**
	**96**	**0.714**	**0.641**	1.252	0.951	0.760	1.729	0.931	0.747	1.937	1.139	0.828	1.296	1.145	0.839	1.374	2.390	1.336	1.279	3.810	1.720	1.189	1.029	0.790	**0.992**
DJI	**12**	**0.128**	**0.245**	**0.385**	0.138	0.258	0.396	0.131	0.251	0.441	0.212	0.360	0.512	0.209	0.359	0.394	2.871	1.561	0.592	2.046	1.311	0.705	0.968	0.825	0.993
	**24**	**0.170**	**0.298**	**0.389**	0.188	0.319	0.455	0.175	0.306	0.474	0.231	0.374	0.411	0.265	0.413	0.471	2.657	1.510	0.635	2.825	1.551	0.778	0.966	0.822	0.995
	**36**	**0.197**	**0.325**	**0.382**	0.214	0.348	0.493	0.200	0.335	0.509	0.315	0.443	0.467	0.319	0.456	0.539	3.183	1.650	0.627	3.080	1.622	0.834	0.965	0.821	1.002
	**96**	**0.261**	**0.391**	**0.448**	0.284	0.415	0.591	0.270	0.403	0.634	0.366	0.484	0.473	0.409	0.522	0.609	3.326	1.691	0.727	3.471	1.721	0.825	0.953	0.812	0.999
HSI	**12**	**0.323**	**0.352**	2.153	0.367	0.378	2.402	0.367	0.372	2.232	0.782	0.701	5.570	0.622	0.604	2.388	0.557	0.513	2.388	0.520	0.474	2.956	0.997	0.799	**0.992**
	**24**	**0.434**	**0.449**	2.559	0.488	0.477	3.247	0.488	0.467	2.981	0.508	0.501	2.794	0.715	0.674	3.394	0.644	0.614	3.394	0.644	0.580	3.185	0.981	0.795	**0.995**
	**36**	**0.561**	**0.519**	2.656	0.563	0.524	3.962	0.586	0.527	3.780	0.590	0.574	3.770	0.832	0.750	2.764	0.726	0.669	2.764	0.809	0.717	4.657	0.968	0.791	**1.001**
	**96**	**0.695**	**0.629**	5.172	0.772	0.666	5.999	0.765	0.651	5.233	0.777	0.704	5.382	1.118	0.900	5.266	0.977	0.808	5.266	1.078	0.877	5.249	0.943	0.783	**0.998**
S&P500	**12**	**0.394**	**0.292**	**0.243**	0.497	0.328	0.281	0.491	0.325	0.300	0.931	0.511	0.420	0.597	0.455	0.377	2.772	1.546	0.578	1.802	1.106	0.444	1.039	0.849	0.990
	**24**	**0.462**	**0.352**	0.229	0.532	0.364	**0.226**	0.614	0.372	0.228	0.463	0.357	0.259	0.624	0.455	0.268	4.622	2.041	0.704	2.778	1.479	0.560	1.039	0.849	0.997
	**36**	**0.447**	**0.343**	**0.164**	0.554	0.381	0.183	0.715	0.397	0.187	0.498	0.391	0.182	0.663	0.461	0.274	4.531	2.015	0.757	3.235	1.633	0.620	1.039	0.848	0.999
	**96**	**0.502**	**0.399**	**0.188**	0.611	0.421	0.207	0.806	0.441	0.202	0.536	0.416	0.196	0.737	0.487	0.244	4.894	2.101	0.823	4.898	2.107	0.802	1.036	0.845	0.997
Count		16	16	7	0	0	1	0	0	0	0	0	1	0	0	0	0	0	1	0	0	0	0	0	8

Optimal avg. metrics are **bolded**; secondary optimal are underlined.

### 4.6 Univariate results

Univariate analysis is based solely on the historical data of a single time series to predict future values. In this experiment, we present the univariate results for four datasets (as shown in Table 3). Compared to other models, our model (CMDMamba) achieved superior performance in the prediction task. The model uses 96 historical data points to predict 58 future data points), our model reduced the Mean Absolute Error (MAE) on the DAX dataset by 4. 2% (0.522 0.500), on the DJI dataset by 8. 7% (0.344 0.314), on the HSI dataset by 1. 5% ( 0.394 0.388), and on the S& P500 dataset by 5. 4% ( 0.185 0.175). Moreover, as the forecasting horizon extends, most baseline models exhibit a noticeable increase in prediction errors–specifically in terms of MSE and MAE–across various datasets. This trend is particularly evident for models such as Autoformer and Informer, which experience significant error escalation at longer time steps. In contrast, our proposed model, CMDMamba, demonstrates comparatively smaller error growth in most scenarios, highlighting its superior stability in long-term forecasting. However, in terms of MAPE, CMDMamba does not exhibit the same level of dominance as observed in multivariate forecasting tasks. This suggests a potential limitation in univariate settings, where limited input information may constrain the model's ability to effectively capture temporal dependencies.

**Table 3:** Univariate forecasting results across four datasets under forecast horizons *O* ∈ {12, 36, 58, 96}.

**Model**		**Ours**			**iTransformer**			**Autoformer**			**Reformer**			**Pyraformer**			**Informer**			**LSTM**			**GARCH**		
**Metric**		**MSE**	**MAE**	**MAPE**	**MSE**	**MAE**	**MAPE**	**MSE**	**MAE**	**MAPE**	**MSE**	**MAE**	**MAPE**	**MSE**	**MAE**	**MAPE**	**MSE**	**MAE**	**MAPE**	**MSE**	**MAE**	**MAPE**	**MSE**	**MAE**	**MAPE**
DAX	**12**	**0.171**	**0.301**	**0.151**	0.188	0.312	0.162	0.751	0.684	0.248	0.809	0.791	0.443	0.271	0.417	0.324	0.489	0.588	0.424	2.280	1.400	0.475	1.026	0.828	0.998
	**24**	**0.338**	**0.431**	0.225	0.348	0.441	**0.213**	0.499	0.561	0.259	1.698	1.189	0.619	0.491	0.565	0.396	0.656	0.676	0.484	3.196	1.684	0.553	1.033	0.832	1.001
	**36**	**0.423**	**0.522**	0.242	0.438	0.500	**0.236**	1.008	0.805	0.339	1.913	1.269	0.659	0.628	0.662	0.504	0.983	0.853	0.505	4.023	1.915	0.612	1.038	0.834	1.005
	**96**	**0.680**	**0.634**	0.265	0.713	0.664	**0.241**	1.245	0.909	0.420	3.249	1.689	0.725	1.524	1.108	0.566	2.656	1.484	0.602	6.505	2.468	0.762	1.053	0.841	1.007
DJI	**12**	**0.064**	**0.182**	**0.063**	0.070	0.188	0.066	0.122	0.270	0.090	2.092	1.392	0.457	1.018	0.935	0.318	1.287	1.060	0.409	1.289	1.073	0.333	0.965	0.832	0.999
	**24**	**0.134**	**0.267**	**0.094**	0.156	0.284	0.103	0.200	0.354	0.125	2.864	1.636	0.547	1.360	1.094	0.400	1.721	1.243	0.485	1.681	1.237	0.382	0.963	0.830	1.000
	**36**	**0.183**	**0.314**	**0.110**	0.215	0.344	0.112	0.350	0.469	0.171	3.743	1.889	0.596	2.101	1.393	0.471	3.036	1.692	0.523	2.166	1.420	0.437	0.962	0.828	1.003
	**96**	**0.261**	**0.382**	**0.133**	0.271	0.388	0.134	0.418	0.515	0.172	4.291	2.028	0.644	3.066	1.705	0.559	3.084	1.703	0.561	2.697	1.561	0.476	0.948	0.818	1.006
HSI	**12**	**0.094**	**0.242**	1.498	0.104	0.255	1.307	0.440	0.529	2.244	0.349	0.444	1.213	0.120	0.256	1.070	0.275	0.402	1.975	0.228	0.371	1.735	0.993	0.815	**0.996**
	**24**	**0.189**	**0.334**	1.581	0.193	0.343	1.480	0.270	0.409	1.920	0.515	0.561	1.488	0.247	0.370	1.683	0.354	0.473	2.288	0.431	0.525	2.583	0.974	0.810	**1.001**
	**36**	**0.245**	**0.388**	2.199	0.255	0.394	2.309	0.633	0.672	3.862	0.695	0.685	1.746	0.364	0.460	2.035	0.649	0.658	2.375	0.664	0.693	3.809	0.959	0.805	**1.002**
	**96**	**0.430**	0.535	3.668	0.435	**0.531**	3.986	0.886	0.815	5.112	0.665	0.660	1.890	0.701	0.666	2.747	0.877	0.726	4.502	0.979	0.892	4.041	0.926	0.796	**1.006**
S&P500	**12**	**0.032**	**0.147**	0.061	0.035	0.148	**0.059**	0.121	0.263	0.130	0.470	0.556	0.247	0.118	0.281	0.087	0.122	0.281	0.207	0.258	0.437	0.157	1.040	0.884	1.000
	**24**	0.052	0.179	**0.073**	**0.048**	**0.170**	**0.073**	0.154	0.317	0.135	1.262	0.993	0.423	0.767	0.771	0.312	0.493	0.594	0.264	0.362	0.522	0.187	1.039	0.882	1.002
	**36**	**0.051**	**0.175**	**0.073**	0.057	0.185	0.079	0.194	0.353	0.150	1.353	1.054	0.508	1.025	0.920	0.351	0.423	0.558	0.344	0.455	0.609	0.221	1.037	0.883	1.006
	**96**	**0.065**	**0.202**	**0.081**	0.080	0.225	0.095	0.169	0.325	0.153	2.221	1.422	0.647	1.538	1.174	0.465	1.223	1.040	0.472	1.756	1.252	0.468	1.029	0.878	1.009
Count		15	13	8	1	3	5	0	0	0	0	0	0	0	0	0	0	0	0	0	0	0	0	0	4

Optimal avg. metrics are **bolded**; secondary optimal are underlined.

### 4.7 Ablation experment

To evaluate the impact of the dual-layer Mamba and DconvFFN on CMDMamba's performance, we conduct an ablation study examining three model variants: tFFN/oMamba, which integrates the DconvFFN module alongside a single Mamba layer; oFFN/tMamba, which excludes the DconvFFN module but incorporates two Mamba layers; and oFFN/oMamba, which omits both the DconvFFN module and Mamba layers. We compare these variants against Reformer and Transformer models across four datasets, utilizing MSE and MAE as evaluation metrics. As presented in Table 4, the results indicate a progressive enhancement in model performance with the inclusion of additional components. This confirms the substantial contribution of both the dual-layer Mamba and DconvFFN module to the overall effectiveness of CMDMamba.

**Table 4:** Ablative experiment results across four datasets under forecast horizons *O* ∈ {12, 36, 58, 96}.

**Model**		**Ours**			**tFFN/oMamba**			**oFFN/tMamba**			**oFFN/oMamba**			**Reformer**			**Transformer**		
**Metric**		**MSE**	**MAE**	**MAPE**	**MSE**	**MAE**	**MAPE**	**MSE**	**MAE**	**MAPE**	**MSE**	**MAE**	**MAPE**	**MSE**	**MAE**	**MAPE**	**MSE**	**MAE**	**MAPE**
DAX	**12**	**0.246**	**0.351**	1.182	0.253	0.357	**1.181**	0.259	0.363	1.315	0.271	0.370	1.429	1.086	0.873	1.450	0.877	0.797	1.363
	**24**	**0.413**	**0.464**	**1.390**	0.422	0.470	1.396	0.422	0.478	1.735	0.420	0.474	1.799	2.106	1.243	1.576	1.153	0.922	1.431
	**36**	**0.504**	**0.520**	**1.321**	0.515	0.527	1.327	0.505	0.528	1.642	0.509	0.529	1.711	2.356	1.322	1.598	1.173	0.919	1.470
	**96**	0.714	0.641	1.452	0.646	**0.602**	**1.449**	**0.643**	0.604	1.650	0.649	0.607	1.736	3.261	1.578	1.684	2.372	1.334	0.549
DJI	**12**	0.128	0.245	0.395	**0.126**	0.244	0.395	0.129	**0.243**	0.391	0.134	0.250	**0.387**	2.001	1.300	0.439	1.309	1.035	0.442
	**24**	**0.170**	**0.298**	0.429	0.175	0.301	0.429	0.176	0.300	0.465	0.179	0.305	**0.455**	2.499	1.457	0.560	1.707	1.214	0.577
	**36**	**0.197**	**0.325**	0.482	0.198	0.326	**0.481**	0.204	0.330	0.504	0.206	0.331	0.488	2.802	1.548	0.558	2.180	1.363	0.536
	**96**	**0.261**	0.391	0.548	0.262	**0.390**	**0.547**	0.266	0.393	0.609	0.264	0.391	0.587	3.562	1.746	0.637	2.463	1.461	0.589
HSI	**12**	**0.323**	0.352	2.530	**0.323**	**0.349**	2.525	0.351	0.359	2.369	0.347	0.359	2.389	0.435	0.440	**1.145**	0.491	0.467	1.395
	**24**	0.434	0.449	3.159	**0.429**	0.448	3.164	0.460	0.457	3.215	0.442	**0.438**	3.277	0.692	0.594	**1.595**	0.522	0.520	1.982
	**36**	0.561	0.519	4.256	**0.517**	**0.502**	4.267	0.565	0.522	3.944	0.549	0.513	4.313	0.875	0.737	**1.454**	0.573	0.547	2.585
	**96**	**0.695**	**0.629**	5.172	0.724	0.648	5.179	0.753	0.654	5.831	0.747	0.653	6.037	0.995	0.763	**2.232**	0.782	0.700	3.443
S&P500	**12**	0.394	0.292	0.243	**0.364**	**0.285**	0.243	0.479	0.323	0.256	0.453	0.322	0.284	1.926	0.799	**0.226**	2.070	0.892	0.277
	**24**	0.462	0.352	0.229	**0.456**	**0.329**	0.229	0.520	0.354	**0.222**	0.520	0.355	0.225	3.104	1.612	0.424	1.793	1.233	0.259
	**36**	**0.447**	**0.343**	**0.164**	0.457	0.348	0.165	0.505	0.358	0.178	0.504	0.362	0.184	3.541	1.734	0.487	2.000	1.281	0.202
	**96**	0.502	0.399	0.198	**0.498**	**0.376**	0.197	0.558	0.402	**0.193**	0.565	0.397	0.208	4.608	2.017	0.634	2.725	1.516	0.218
Count		9	7	3	7	7	4	1	1	2	0	1	2	0	0	5	0	0	0

Optimal avg. metrics are **bolded**; secondary optimal are underlined.

## 5 Discussion

### 5.1 Hyper-parameter sensitivity analysis

To rigorously assess the parameter sensitivity of CMDMamba, we systematically examined two pivotal hyperparameters through cross-market evaluations using datasets representing diverse economic environments: (a) dropout rate and (b) SSM state expansion factor. Our experimental protocol maintained fixed non-target parameters while executing five independent training runs per configuration, each spanning 30 epochs with early stopping monitoring validation loss (patience threshold = 5 epochs). Model performance was rigorously evaluated using MSE metrics across four distinct forecast horizons O∈{12,36,58,96}.. This approach aligns with the rigorous evaluation methodologies used in recent studies on hyperparameter tuning for deep learning models in time series forecasting, ensuring that our findings are comparable and robust.

#### 5.1.1 Dropout

**Figure 4:**
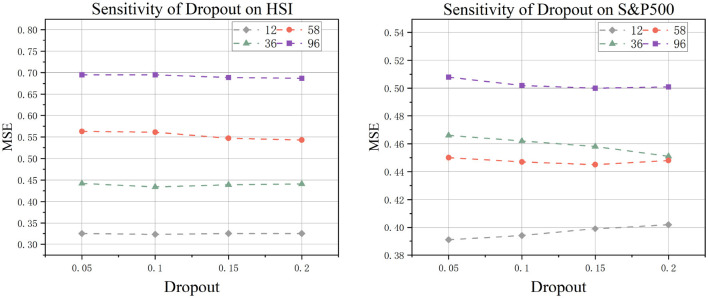
Experiment on the sensitivity of model to dropout.

In our architecture, the dropout mechanism is implemented within the DconvFFN layers (as illustrated in Figure 4). This technique alleviates overfitting and enhances model generalization by randomly zeroing out each element of the input tensor with probability *p*. Systematic comparisons of dropout rates *p* ∈ {0.05, 0.1, 0.15, 0.2} demonstrate that the model achieves optimal overall performance at *p* = 0.1. The results indicate that insufficient regularization occurs with excessively low dropout rates, while overly high dropout rates degrade performance by impairing the representational capacity of the deep convolutional feed-forward network.

#### 5.1.2 SSM state expansion factor

**Figure 5:**
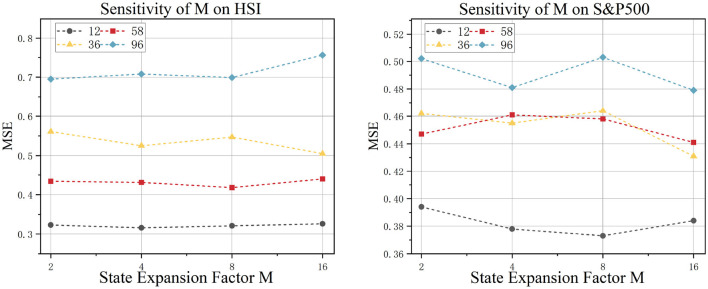
Model sensitivity to SSM state expansion factor experiment.

This study examines the impact of the expansion factor *M* in state space models on time series forecasting performance. The experimental results are shown in Figure 5. When *M* ∈ {2, 4, 8, 16}, distinct patterns emerge: In the S&P500 dataset, which represents global multi-market dynamics, forecasting accuracy significantly improves as *M* increases. This confirms that cross-market linkage effects require higher-dimensional latent states for effective capture. Conversely, in the regionally focused HSI (Hang Seng Index) dataset, variations in *M* have almost no impact. This suggests that its endogenous temporal patterns exhibit low-dimensional separability, where excessive expansion of the state dimension may introduce noise interference.

### 5.2 Generalization and predictive insights of the model on stock values

**Figure 6:**
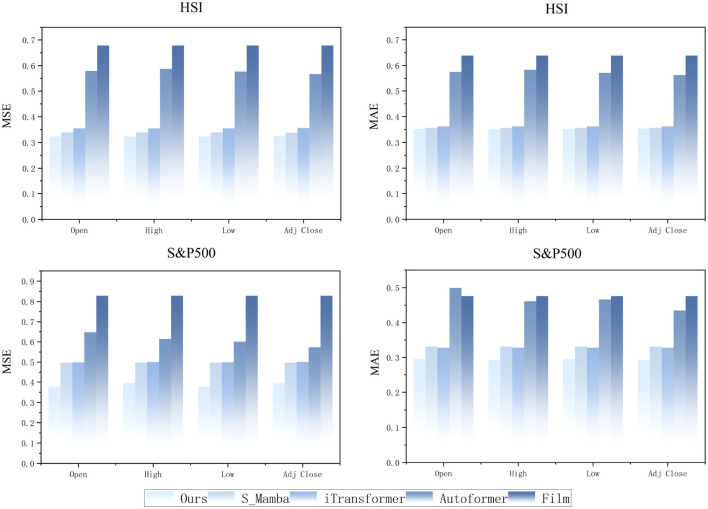
Prediction results of stock price variables by different models with input length *L* = 96 and forecast horizon *O* = 12.

Based on the high accuracy demonstrated by the CMDMamba model in predicting closing prices (Close), we further evaluated its generalization capability through experiments on two distinct market economy datasets: the regional HSI and the globally representative S&P500 Index. Our study extended the model's application to four additional crucial price variables—opening price (Open), highest price (High), lowest price (Low), and adjusted closing price (Adj Close)—which represent various aspects of financial market behavior. As illustrated in Figure 6, the CMDMamba model exhibits superior performance in these diverse stock price prediction tasks.

### 5.3 Model efficiency

**Figure 7:**
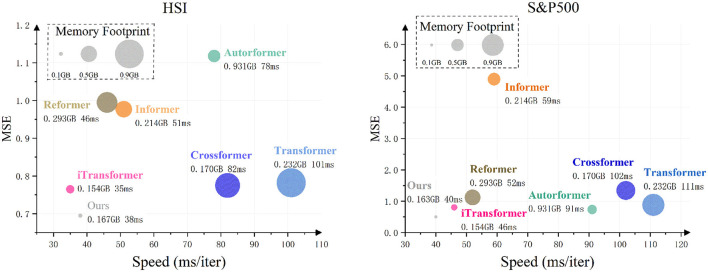
Comparison of model efficiency on HSI and S&P500 datasets with sequence length *L* = 96 and forecast horizon *O* = 12, using a batch size of 12.

In this experiment, we comprehensively evaluated model efficiency from three dimensions: (a) predictive accuracy, (b) memory usage, and (c) training speed. For the prediction task, we set *L* = 96 (input length), *O* = 12 (forecast horizon), and *B* = 8 (batch size), with results presented in Figure 7. All other parameters were fixed to ensure modeling consistency. A direct comparison was made between the self-attention mechanism and CMDMamba. In HSI data tests focusing on regional markets, iTransformer demonstrated marginally superior computational efficiency compared to CMDMamba. However, CMDMamba achieved better prediction performance and memory optimization, showing particular effectiveness in capturing temporal patterns in relatively stable sequences. For the S&P500 dataset representing global multi-market dynamics, both CMDMamba and self-attention-based models exhibited strong predictive capabilities in volatile market conditions. Notably, CMDMamba outperformed all counterparts across both datasets. This advantage stems from the linear complexity characteristics of SSM, enabling CMDMamba to achieve an optimal balance between prediction accuracy, training speed, and memory consumption. This finding is consistent with recent advancements in efficient sequence modeling, where linear complexity models are increasingly preferred over quadratic complexity models for large-scale applications. Overall, compared to self-attention mechanisms in Transformers with equivalent embedding dimension *D*, CMDMamba demonstrates significantly lower computational costs, particularly evident in training speed and GPU memory utilization. With superior predictive performance, accelerated training efficiency, and reduced memory requirements, Mamba-like models exhibit great potential for financial time series forecasting applications.

### 5.4 Performance under different input sequences

**Figure 8:**
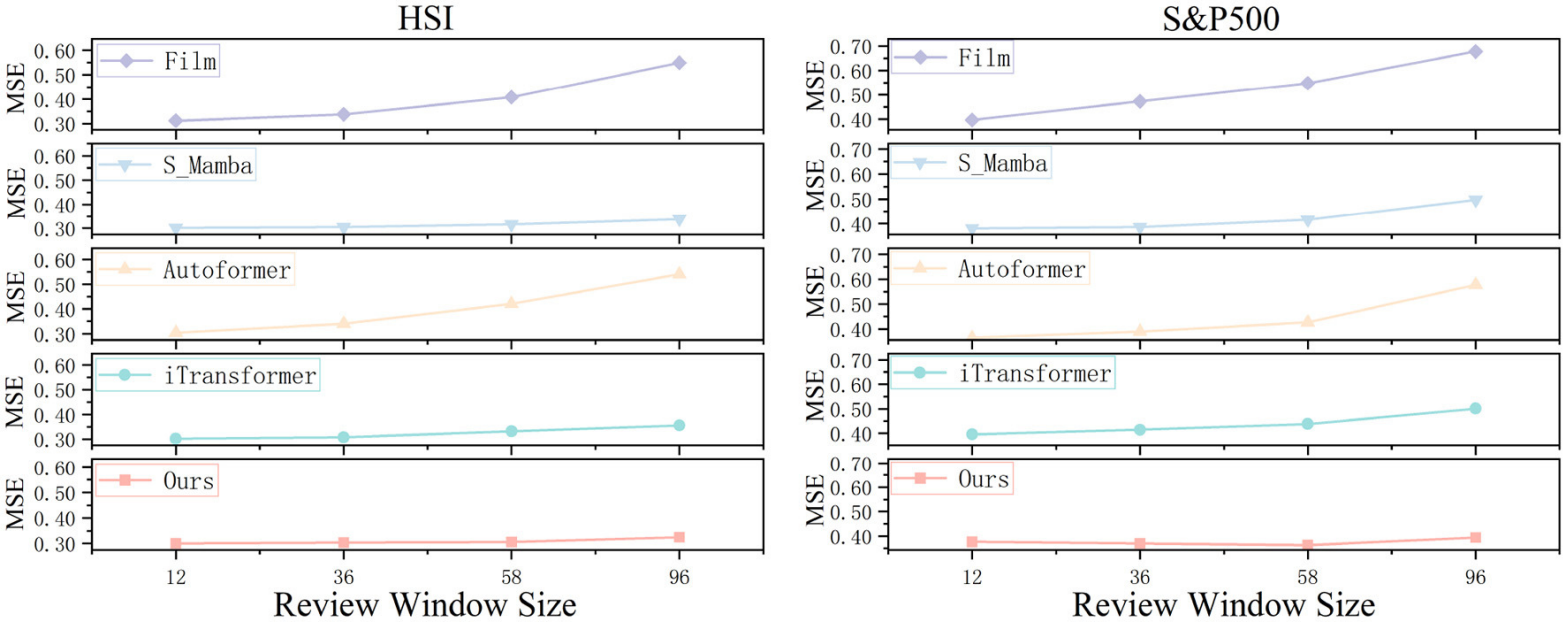
Predicted line graphs of two datasets across different review windows. We compare these with four other models.

The selection of an appropriate review window size is critical for time series forecasting models, as it determines the extent to which historical dependencies can be captured. A proficient forecasting model should be capable of learning long-term dependencies by expanding the review window, thereby enhancing predictive accuracy. In our experimental design, we maintained a fixed forecast horizon (= 12) across two heterogeneous datasets while systematically varying the review window sizes ( ∈ {12, 36, 58, 96}), ensuring that all other model parameters remained constant to facilitate a reliable comparative analysis. As illustrated in Figure 8, the CMDMamba model consistently outperforms across all review window configurations, demonstrating its robust forecasting capability. This empirical advantage can be attributed to two key architectural innovations. First, the DconvFFN module efficiently captures multivariate feature dependencies by leveraging dilated convolutional operations. Second, the dual-layer Mamba architecture integrates differentially sensitized modules: the shallower layer specializes in detecting local fluctuations within shorter review windows through high-frequency filtering, while the deeper layer focuses on extracting global dependencies in extended sequences via low-frequency pattern analysis. These design principles collectively enable CMDMamba to achieve superior forecasting performance across diverse time series scenarios.

### 5.5 Comparison of different deep learning models

**Figure 9:**
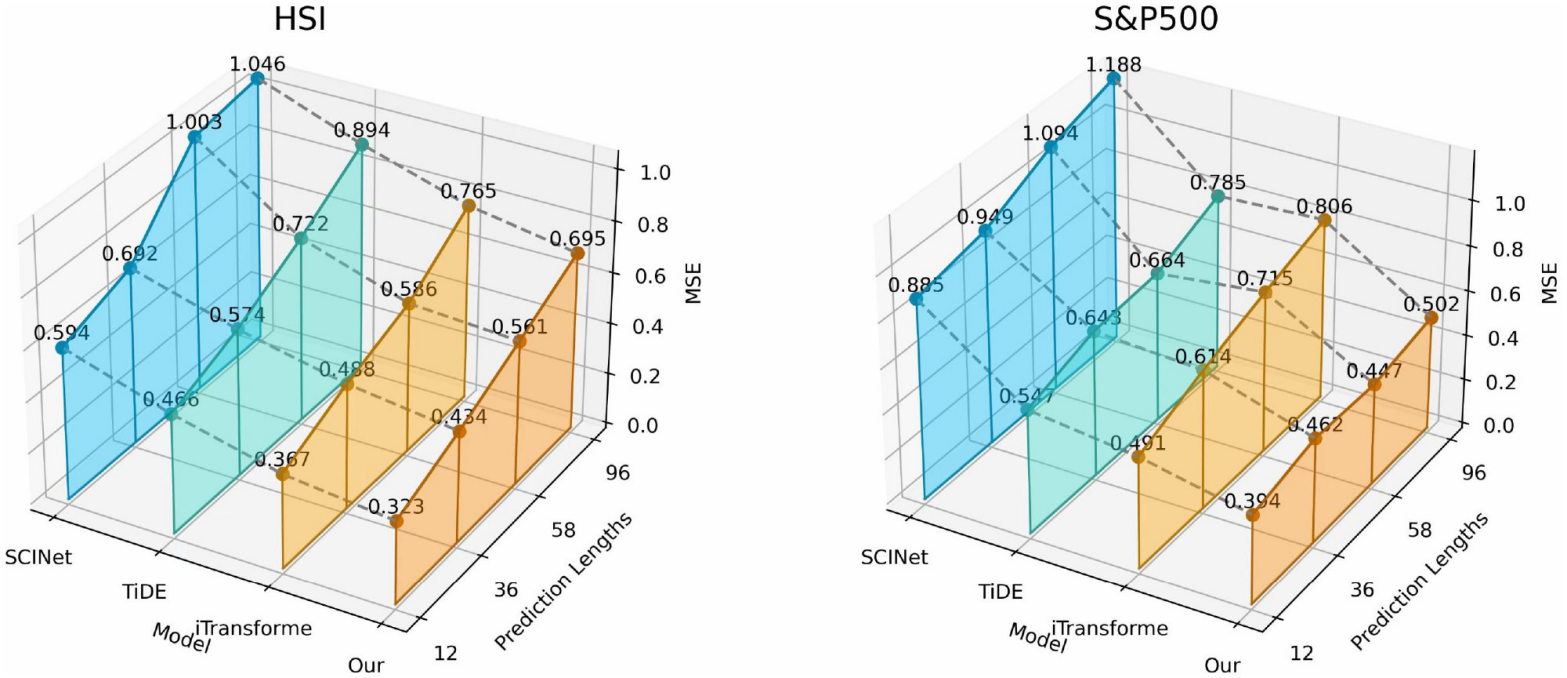
3D graphs of different deep learning frameworks on two datasets, presenting the results achieved for different forecast horizons *O* ∈ {12, 36, 58, 96}.

In this study, we systematically evaluated the performance of four mainstream architectures–state-space models, Transformer, temporal convolution, and MLP–in financial time series forecasting using datasets such as HSI and S&P500. As shown in Figure 9, compared with the iTransformer, which also has the ability to model global dependencies, the CMDMamba based on Mamba not only demonstrates superior predictive performance but also optimizes the computational complexity to linear. When compared with SCINet, which has a convolutional module, CMDMamba can better capture global dependencies through its selective attention mechanism, showing significant performance advantages over temporal convolutional models. Compared with the MLP-based TiDE, CMDMamba employs a dual-layer Mamba framework that can adaptively enhance attention weights for recent abnormal fluctuations, while the static processing mode of the MLP lacks flexibility in responding to market dynamics. These findings indicate that Mamba, as an emerging deep learning framework, shows great potential and broad application prospects in financial sequence prediction by balancing computational efficiency and the ability to model global dependencies.

### 5.6 Model's adaptability to noise

**Figure 10:**
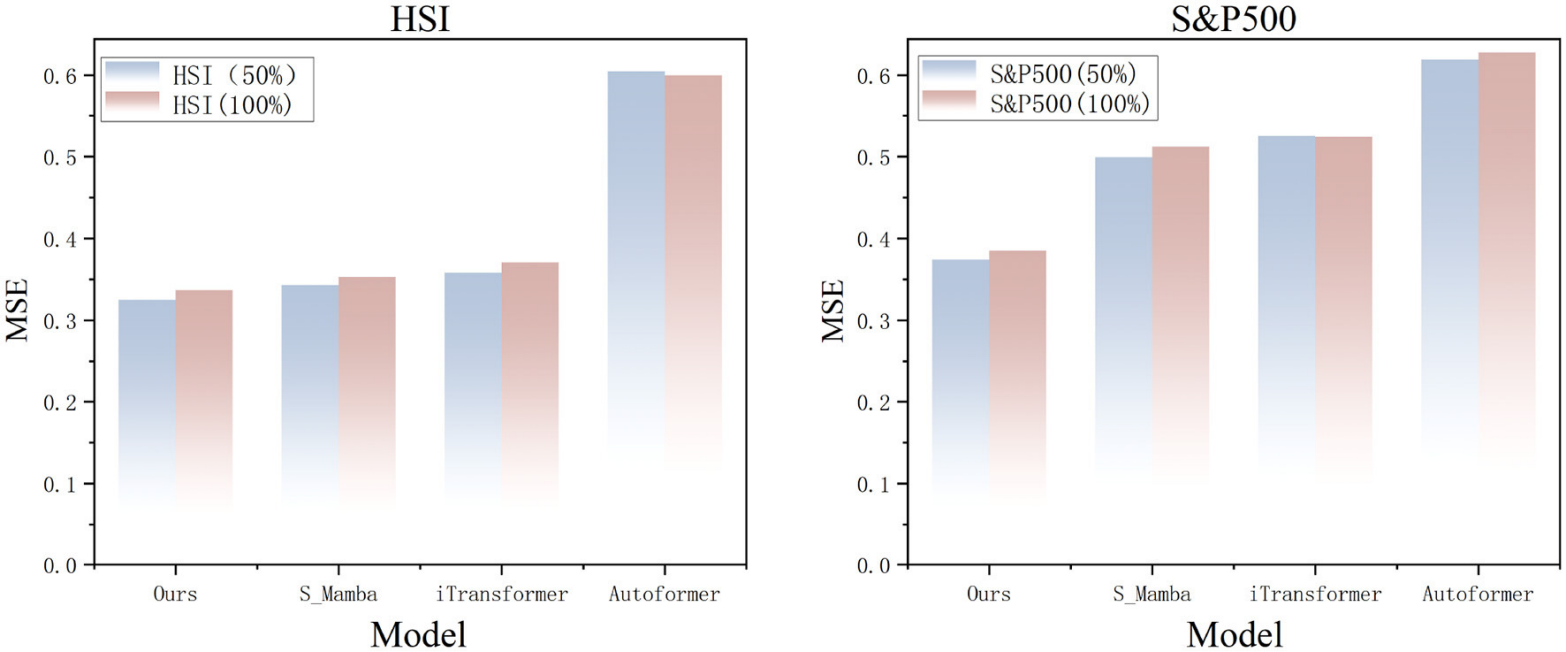
Prediction results reveal the noise resistance performance of different models on HSI and S&P500 datasets with input length *L* = 96 and forecast horizon *O* = 12.

This study systematically evaluates the robustness of the CMDMamba model against noise interference in financial time series data, addressing the pervasive challenges of multidimensional game noise and market information friction. Experiments applied 50% and 100% Gaussian white noise to the HSI and S&P500 datasets, simulating variations in the signal-to-noise ratio (SNR) in real-world markets. The results (see Figure 10) show that CMDMamba outperforms Transformer-based architectures in both dynamic noise testing and cross-market validation. The model's dual-layer Mamba mechanism can effectively suppress noise while maintaining linear computational complexity. Compared to self-attention mechanisms, this approach reduces redundancy and achieves precise noise-signal separation in complex financial environments.

### 5.7 Impact analysis of price features on model performance

**Figure 11:**
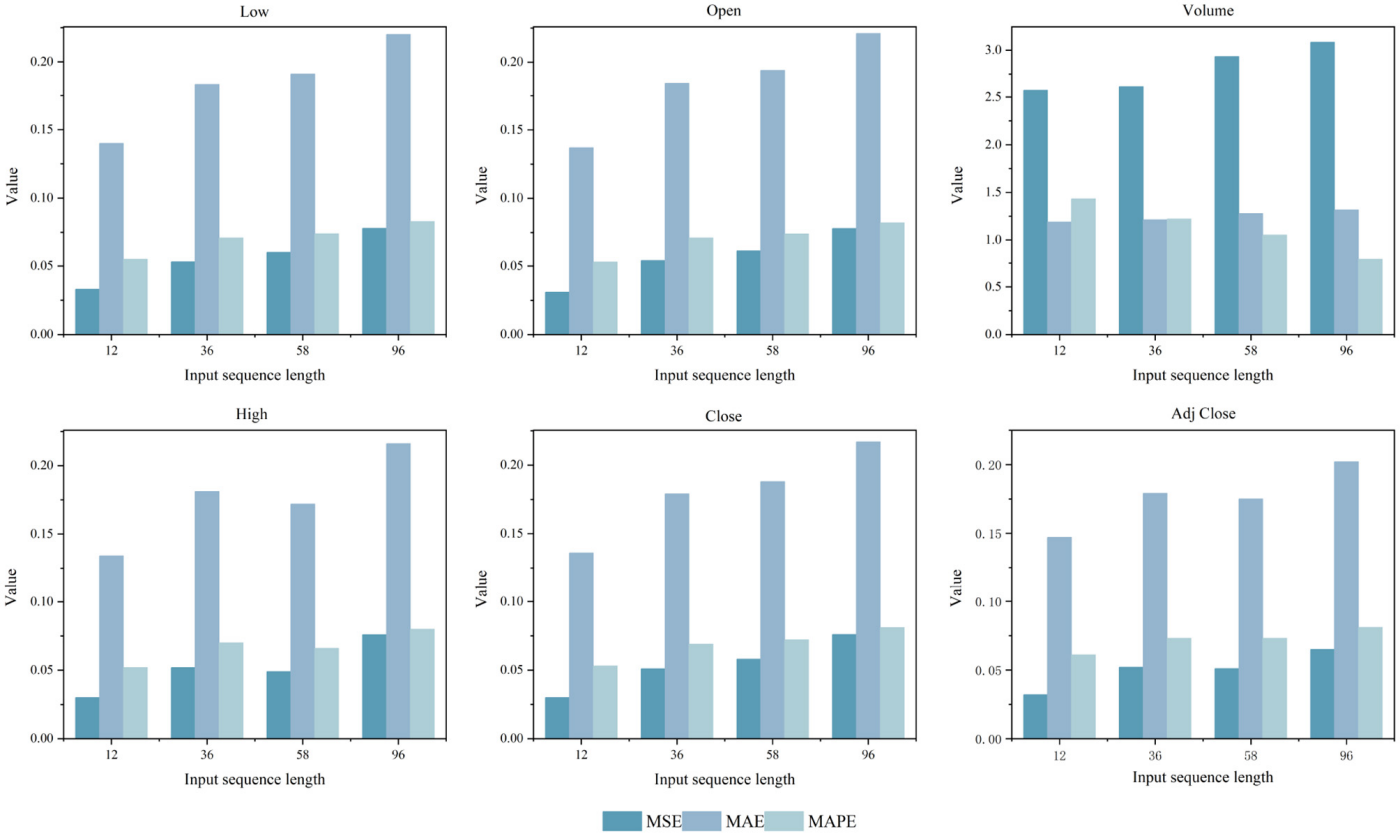
Comparison results of MSE, MAE, and MAPE for five key price indicators and trading volume across four different sequence length *L* ∈ {12, 36, 58, 96}.

This study employs a univariate forecasting approach to systematically assess the impact of six key financial indicators on model performance across varying input sequence lengths *L* ∈ {12, 36, 58, 96}, as shown in Figure 11. The results indicate that the five price-related indicators demonstrate relatively consistent prediction accuracy and exhibit limited sensitivity to changes in input length. In contrast, trading volume presents significant variability. Owing to its high trading activity, pronounced volatility, and the absence of clear trends or structural stability, its prediction errors are notably sensitive to input sequence variations. Furthermore, to address the high degree of correlation among price-based features, each variable was modeled independently to evaluate changes in model performance when using only a single price indicator.

## 6 Conclusions

In this study, we propose CMDMamba, an innovative dual-layer Mamba-based model that demonstrates exceptional hierarchical learning capabilities. The high-sensitivity branch precisely captures subtle fluctuations in financial markets, while the low-sensitivity branch focuses on modeling macro-trend dynamics. Leveraging Mamba's linear computational complexity, our framework significantly reduces operational costs without compromising performance. The DConvFFN module further enhances feature representation through temporal depth-wise convolution and cross-variable point-wise operations, achieving dynamic noise suppression and efficient extraction of multi-scale temporal patterns. Through comprehensive comparisons with various deep learning frameworks, we validate the potential of Mamba-based architectures for time series forecasting tasks.

Looking forward, future research will expand into multi-domain applications, particularly focusing on datasets with irregular patterns.CMDMamba not only overcomes the limitations of existing models in capturing the complexities of financial time series, but also delivers a more efficient and accurate solution for forecasting. With its innovative dual-layer Mamba architecture and DconvFFN module, CMDMamba achieves significantly improved prediction accuracy while preserving linear computational complexity. These advancements provide both a solid theoretical foundation and practical insights for future research and applications in financial time series forecasting. We are confident that continued optimization and functional enhancements will enable CMDMamba to achieve more remarkable results in cross-domain time series prediction. In summary, CMDMamba represents a significant advancement in financial time series forecasting. These findings not only underscore its robust efficacy but also highlight its substantial potential and broad application prospects in this critical field, laying a solid foundation for subsequent research and practical implementations.

## Data Availability

The datasets presented in this study can be found in online repositories. The names of the repository/repositories and accession number(s) can be found in the article/supplementary material.
